# Internal migration and child health: An investigation of health disparities between migrant children and left-behind children in China

**DOI:** 10.1371/journal.pone.0265407

**Published:** 2022-03-16

**Authors:** Yue Zhang, Xiaodong Zheng

**Affiliations:** School of Economics, Zhejiang Gongshang University, Hangzhou, Zhejiang, China; Flinders University, AUSTRALIA

## Abstract

Using data from the China Education Panel Survey (CEPS), this study empirically examines the association between internal migration and child health through an investigation of health disparities between migrant children and left-behind children in China. The results show that, in comparison with being left behind, migrating with parents significantly improves children’s self-reported health, height-for-age z-score (HAZ) and BMI-for-age z-score (BAZ), and reduces their frequency of sickness. These findings remain robust to a suite of robustness checks. Furthermore, the health effects of internal migration are more prominent for children with a rural *hukou* compared with urban ones. Although migrant children are more likely to experience teacher discrimination, they have higher levels of parental care, family relationships, and peer relationships relative to their left-behind counterparts, which indicates possible mechanisms behind the association between children’s migration and health. Our findings underline the importance of policy improvement and evidence-based interventions aiming at reducing involuntary parent-child separation and facilitating the development in health of disadvantaged children in developing countries like China.

## Introduction

Due to the growing scope and impacts of population movements, migration is becoming one of the determining global phenomena of the 21st century [1]. As the largest developing country in the world, the massive internal migration has greatly facilitated China’s urbanization and economic development over the past decades. However, the household registration (*hukou*) system, which is often associated with the receipt of local programs and benefits such as education, health care and social security, has long been the main institutional barrier for Chinese internal migrants to bring their family members (such as their children) to the destination of migration [2]. As a result, a large number of children have either been migrated with their parents or left behind at home while parents moving away for work [3, 4]. According to the statistics released by the Ministry of Education of China, there were 14.27 million migrant children and 13.84 million rural left-behind children in China by 2019 [5]. Migration not only represents geographical movements of residence but also has profound impacts on the well-being of migrants and their children [6]. Given that children’s growth and development are crucial to achieving their full potential as healthy adults later in life, the health issues of migrant and left-behind children have been widely concerned for policymakers and academia [7, 8]. There has been substantial evidence on the effects of parental migration on the nutrition and health of left-behind children (LBC), using local non-left-behind children as the reference group [9–13]. Meanwhile, many previous studies have also examined the health differences between migrant children and urban native children [14, 15]. Nonetheless, empirical evidence regarding the health disparities between migrant children and their left-behind counterparts remains limited. This gap in the literature is especially important because policy interventions for children of migrants could be ill-targeted without identifying the relatively vulnerable children during the process of internal migration. As such, investigating the health disparities between migrant children and left-behind children can help improve relevant policies to promote the development of disadvantaged children and reduce social inequality in the long run.

Existing research often investigates the health issues of internal migrants’ children from the perspective of comparing the differences in health outcomes between children of concern and their local peers (i.e., migrant children vs. urban native children; left-behind children vs local non-left-behind children). In terms of migrant children, it has been demonstrated that they are often confronted with the problems of social exclusion and discrimination induced by the local social environment or institutional barriers, particularly the *hukou* system [6]. For example, existing evidence has demonstrated that a non-negligible proportion of migrant children without an urban *hukou* cannot enjoy the same educational opportunity and quality as local children [16]. As a result, these children are enrolled in special migrant schools rather than local public schools [17]. Meanwhile, migrant children may encounter prejudice and even discrimination within schools, such as discriminative teachers [18]. Previous studies have shown that the perception of discrimination is negatively associated with children’s health, especially migrant children’s psychological well-being [19]. In addition, migrant children may also have to face the challenges to adapt new environment with different social rules and values [15]. As such, compared with their urban native peers, migrant children are not only disadvantaged by household socioeconomic status but also the external social, cultural, and institutional environment [20]. A growing number of studies have suggested that migrant children have lower levels of health and subjective well-being in comparison with local children [21, 22].

With respect to left-behind children, past research has documented that parental migration affects their health mainly through income effect and separation effect (or time effect) [23, 24]. On the one hand, parental migration increases household income and yields remittances to the caregivers of children in home communities, which can subsequently improve the nutritional intake and medical care of left-behind children due to increased health investments [10, 25]. Migrants may also acquire more health knowledge from their peers in cities to better foster the growth and development of their children if they return home regularly [20]. On the other hand, parental migration also means parent-child separation, which can result in a lack of parental care, supervision, and protection. Previous studies have demonstrated that parental absence increases the risks of children’s malnutrition, chronic diseases, and health risk behaviors [10–12]. Compared with their non-left-behind peers living with parents, left-behind children are also shown to be more likely to experience bullying perpetration, accidents, and injuries [26, 27]. To date, although there is still no consensus on the health consequence of parental migration, most empirical studies have suggested that left-behind children have poorer health than non-left-behind ones [28].

Overall, most previous studies have suggested that both migrant and left-behind children are more vulnerable in health compared with their local peers. But few empirical studies have investigated the health differences between these two groups of children. Theoretically, although migrant children may confront challenges of acculturation of a new living environment and social exclusion, they often have higher levels of parental care and family relationships and are less likely to experience health and behavioral problems triggered by parent-child separation [13, 29]. With parental care and protection, migrating with parents also provides an opportunity for children to broaden their horizons, make new friends, and maintain healthy peer relationships, which are crucial for children’s social, behavioral, and health development [20]. Furthermore, it has been reported that official policies highlighted the importance of social integration of migrant households and the equality of developmental opportunity among migrant children in recent years [17]. Consequently, social exclusion and discrimination are not likely to play a major role in health disparities between migrant children and left-behind children. Hence, we assumed that migrating with parents was linked to better health of children compared with being left behind. In addition, in comparison with urban-to-urban migration, the distance and duration of rural-to-urban migration are often longer due to more inter-provincial movements [16]. Urban children are also more likely to be exposed to resource-rich neighborhoods and schools, social security, and protection (e.g., medical insurance) than rural children [30]. As such, we assumed that the health differences between migrating with parents and being left behind would be more pronounced among rural children.

Using data from the China Educational Panel Survey (CEPS), the purpose of this study was to examine the health consequences of children’s migration in China. Different from most past research, this study analyzed the health differences between migrant children and left-behind children, which are the two types of children of internal migrants. Furthermore, we investigated the urban-rural heterogeneity and possible mechanisms of the health disparities between migrant children and left-behind children. Specifically, the potential pathways that were examined in this study included parental care, family relationships, peer relationships, and teacher discrimination. In general, our study contributes to the literature on the health consequences of internal migration and corresponding mechanisms from the perspective of heterogeneity between migrant children and left-behind children. The results can help identify the disadvantaged children more clearly during the process of internal migration in China. Our findings are also beneficial to inform future policies to optimize the allocation of public resources for the development of children of migrants in China and other contexts with a large-scale of migrants. According to our theoretical analysis, we proposed three hypotheses as follows:

*Hypothesis 1 (H1)*: Compared with being left behind, migrating with parents is helpful to improve children’s health.
*Hypothesis 2 (H2)*: The health disparities between migrant children and left-behind children are more prominent among rural children than urban ones.
*Hypothesis 3 (H3)*: Parental care, family relationship, and peer relationship play a major role in the mechanisms underlying the health differences between migrant children and left-behind children.

## Data and methods

### Data and variables

Data used in this study were drawn from the China Education Panel Survey (CEPS). The CEPS is a nationally representative longitudinal survey for junior high school students, which applies a stratified and multistage sampling design with probability proportional to size (PPS). In the academic year 2014~2015, a total of 9,449 8th grade students in 112 junior high schools were interviewed. We restricted our sample to children of the internal migrants and the final sample size was 2,780. Among them, students (living with their parents) whose *hukou* registration place was not their residential location were regarded as migrant children (*N* = 1,348, 48.5%). Left-behind children (*N* = 1,432, 51.5%) were identified as students who lived in the *hukou* registration place while their parents moved away to other counties or provinces.

Based on the available information regarding health in the CEPS data, this study utilized four indicators to measure child health. First, we used self-reported health (very unhealthy = 1 to very healthy = 5) as an overall health measure. Second, the frequency of sickness, such as cold, fever, cough, diarrhea, in the past year (never = 1, seldom = 2, often = 3) was applied as a proxy indicator of physical health. Third, to assess nutritional status, we used two anthropometric measures including height-for-age z-score (HAZ) and BMI-for-age z-score (BAZ). HAZ and BAZ indicators are widely accepted objective measures of child health [31, 32]. They are often used to determine whether a child is growing “normally” or has a growth problem according to the height and Body Mass Index (BMI) of children and corresponding child growth standards. It has been proved that these child health indicators are important predictors of the short- and long-term health of children [33]. HAZ and BAZ are comparable across different genders and ages. The derivation of the two anthropometric measures is: $Z_{i}={(y}_{ij}-\overset{-}{y_{\dot{j}}})/\sigma_{j}$, where *y*_*i*_ is the height (cm) or BMI (kg/m^2^) of child *i* in group *j*. Group *j* is defined by gender and age (in months). $\overset{-}{y_{\dot{j}}}$ and *σ*_*j*_ are the mean and standard deviation of the height (cm) or BMI of the reference group *j* of the same gender and age according to World Health Organization (WHO) child growth standards [34]. Higher values of HAZ and BAZ often indicate a better nutritional status of children.

In addition, we controlled for characteristics of children and their families to reduce potential confounding effects in our empirical analysis. First, given the different growth statuses among different sexes and ages of children, child gender (male = 1) and age (in years) variables were included as controls in our study. Second, existing evidence has shown that children with an urban *hukou* had significantly better health than children with a rural *hukou* because China’s *hukou* system determines eligibility for various welfare benefits [35]. Thus, children’s *hukou* status (rural = 1) was also treated as a control variable in our regression analysis. Third, it is reported that the number of siblings is also an important determinant for children’s physical development [36]. According to the quantity-quality trade-off theory, only children often have more nutrition intake than children with siblings because of more parental investments and care [37]. As such, the only child (yes = 1) status of children was also controlled when investigating the health disparities between migrant children and left-behind children. Fourth, we also controlled for children’s school boarding (yes = 1) status since past research suggested that boarding was often negatively associated with children’s nutrition and health [38, 39]. Finally, it is also well-documented that high family socioeconomic status is critical for a good parenting style and healthy living environment, which can subsequently impact child health [40, 41]. Therefore, as two crucial measures of household socioeconomic status (SES), family economic status (poor = 1 to very rich = 4), and parental education (parental average educational years) were also treated as controls in our empirical analysis.

### Empirical strategy

The baseline regressions for our empirical analysis are specified as follows:

$$h_{ij}=\beta_{0i}+\beta_{1i}{Migrant}_{j}+X_{j}\lambda_{i}+\varepsilon_{ij}$$ (1)

where *h*_*ij*_ is the *i*th health outcome for *j*th child, *Migrant*_*j*_ is a dummy variable indicating the migrant status of the *j*th child, ***X***_*j*_ represents a vector of control variables, and *ε*_*ij*_ denotes the error term. In baseline regressions, we applied ordered probit model for ordinal outcome variables and ordinary least square (OLS) estimation for continuous dependent variables. To account for potential self-selection bias, we further employed propensity score matching (PSM) and inverse probability weighting with regression adjustment (IPWRA). Both the PSM and IPWRA approaches firstly estimate the selection to treatment (*Migrant* = 1) and predict the probabilities of being treated (propensity scores) for all observations. We used a binary probit model to estimate the treatment model as follows:

$$\text{Pr}\left({Migrant}_{j}=1\right)=F(X_{j};\alpha )+\mu_{j}$$ (2)

Then, the PSM approach matches the treated and control groups and compares the differences in outcomes based on propensity scores directly, while the IPWRA method assigns the inverse of probability for the treated and control groups, and re-estimates Eq (1) using new weights [42]. To address potential omitted variable bias, we conducted two sensitivity tests. First, we performed Oster’s bounds analysis to test the sensitivity of OLS estimates to the unobservables based on the selection of observables [43]. The basic idea of Oster’s bounding lies in the proportional selection assumption. The proportional selection assumption indicates that determinants of child health consist of both observed and unobserved factors, and none of them could dominate children’s health status. Therefore, if the observables are randomly chosen from all determinants of child health, then one can calculate reasonable bounds to examine the coefficient stability on the basis of informativeness of the selected observables, which are proportional to the unobservables [44]. Second, we calculated Rosenbaum bounds for the PSM estimates in the presence of unobserved heterogeneity [45]. The key concept of Rosenbaum bounding analysis is to see how much the estimated treatment effects may vary if the estimated odds of migrating with parents (vs. being left-behind) are manipulated by different levels of hidden bias. Additionally, to alleviate the concern of possible reverse causality problem, we used children’s migrant status in 2013~2014 (lagged key independent variable) to identify migrant and left-behind children and re-estimate the baseline regressions.

## Results

### Descriptive statistics

Table 1 presents the summary characteristics of our sample. Overall, the average age of sample children was 14.63 years old. Among them, 48.5% of the respondents were migrant children, 54% were males, 61.9% had a rural *hukou*, 30% were in a boarding school, and 35% were the only child in the household. compared with left-behind children, migrant children had higher self-reported health, HAZ and BAZ, and a lower frequency of sickness, which preliminarily indicates that migrant children were healthier than left-behind children. In addition, there were also significant differences in some individual and family characteristics between the two groups of children (e.g., boarding status, only child status, family economic status, and parental education), implying that children’s migrant status was not random and the self-selection issue should be considered in the empirical analysis.

**Table 1:** The summary statistics of variables.

Variables	Migrant children (Yes = 1)				
	All	Yes	No	Mean Diff.	Sig.
	(1)	(2)	(3)	(4)	(5)
Self-reported health	3.778	3.885	3.667	0.218	***
Sick frequency	1.961	1.932	1.990	-0.058	***
Height for age	0.018	0.128	-0.092	0.220	***
BMI for age	-0.378	-0.250	-0.501	0.251	***
Migrant children	0.485	—	—	—	—
Male	0.540	0.547	0.534	0.013	NS
Age	14.626	14.607	14.645	-0.038	NS
Rural *hukou*	0.619	0.617	0.622	-0.005	NS
Boarding	0.300	0.177	0.424	-0.247	***
Only child	0.350	0.322	0.360	-0.038	**
Family economic status	2.877	2.993	2.753	0.240	***
Parental education	9.611	9.709	9.404	0.305	***

*Notes*: Descriptive statistics (mean characteristics) are shown for the total sample and those who were migrant children compared with those who were left behind. Column (4) and column (5) report mean differences of the variables between the two groups and the corresponding statistical significance levels from *t*-tests, respectively. “NS” denotes not significant.

* *p* < 0.1,

** *p* < 0.05,

*** *p* < 0.01.

### Internal migration and health among migrant and left-behind children

**Fig 1:**
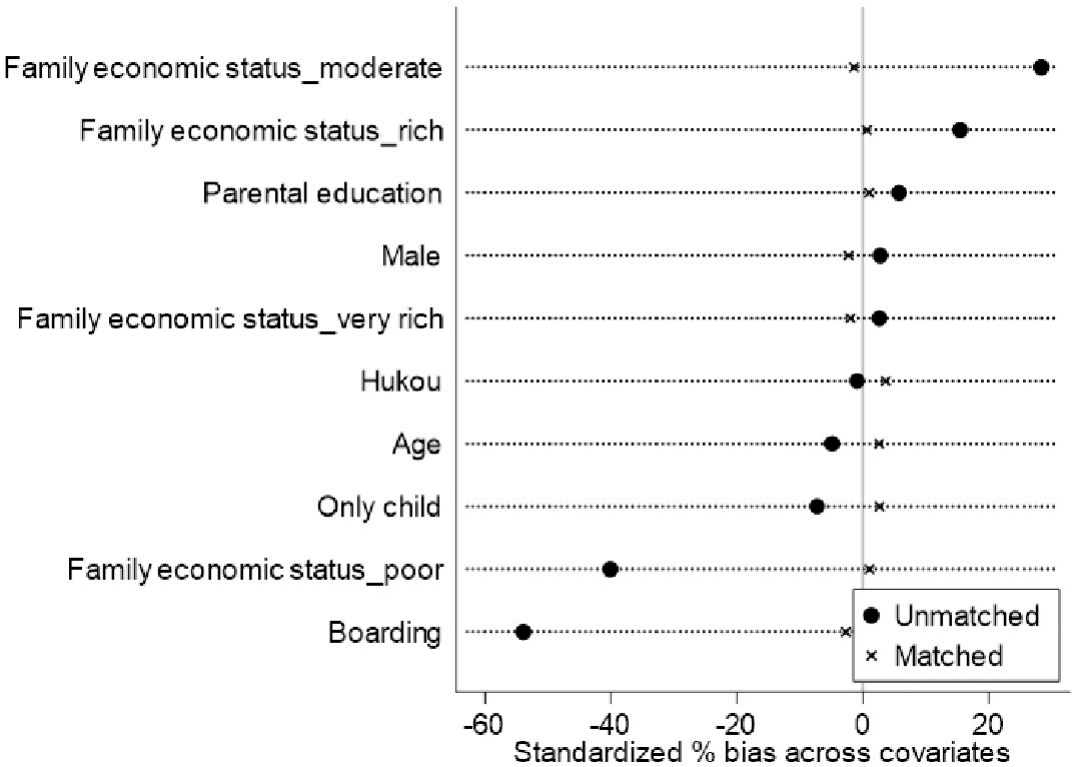
Propensity distributions of treated and control groups before and after matching.

Table 2 presents the estimates of baseline regressions (Panel A) and a series of robustness checks (Panel B to Panel D). In Panel A, the ordered probit and OLS estimates showed that migrating with parents significantly and positively predicted children’s self-reported health (β = 0.198, *p<* 0.01), while it was negatively associated with sick frequency (β = -0.100, *p<* 0.05). Holding the control variables fixed, migrant children also significantly outperformed their left-behind counterparts in HAZ and BAZ by 0.155 (*p<* 0.01) and 0.186 (*p<* 0.01) standard deviations, respectively. In Panel B, the estimates of the three PSM approaches, including nearest neighbor matching (k = 1, one-to-one matching with no replacement), radius matching (r = 0.05), and kernel matching, consistently indicated significantly positive associations of internal migration with children’s self-reported health, HAZ and BAZ, as well as a negative relationship between children’s migration and their sick frequency. Fig 1 and Table 3 demonstrate the balance of the characteristics between migrant and left-behind children before and after PSM estimation. The results revealed that some characteristics were significantly different between the two groups of children before matching, such as boarding status and family economic status. Nonetheless, all of the covariates between migrant and left-behind children were no longer significant and the standardized biases across covariates were close to zero after matching, indicating that the observed self-selection bias has been largely corrected. In Panel C, the IPWRA estimates also coincidentally suggested that children’s migration positively predicted their health, including higher levels of self-reported health, HAZ and BAZ, and lower frequency of sickness. In addition, we further used children’s migrant status in academic year 2013~2014 as the key independent variable and re-estimated the baseline regressions. As shown in Panel D, the results were also robust when using the migrant status in 2013~2014 (lagged independent variable) to measure children’s migration, indicating that reverse causality should not be a serious issue in our study.

**Table 2:** Estimates of health disparities between migrant children and left-behind children.

	(1)	(2)	(3)	(4)
	Self-reported health	Sick frequency	HAZ	BAZ
*Panel A*: *Ordered probit / OLS*				
Migrant children	0.198***	-0.100**	0.155***	0.186***
	(0.046)	(0.047)	(0.050)	(0.059)
*Panel B*: *PSM*				
Nearest neighbor matching (k = 1)	0.223***	-0.055**	0.172***	0.332***
	(0.047)	(0.024)	(0.054)	(0.075)
Radius matching (r = 0.05)	0.180***	-0.036*	0.156***	0.249***
	(0.040)	(0.020)	(0.044)	(0.063)
Kernel matching	0.179***	-0.036*	0.159***	0.252***
	(0.040)	(0.020)	(0.043)	(0.063)
*Panel C*: *IPWRA*				
Migrant children	0.177***	-0.034*	0.178***	0.191***
	(0.037)	(0.018)	(0.039)	(0.056)
*N*	2759	2757	2728	2678
*Panel D*: *Migrant status in 2013~2014*				
Migrant children	0.210***	-0.110**	0.235***	0.169**
	(0.053)	(0.054)	(0.056)	(0.067)
*N*	2201	2202	2182	2142

*Notes*: All control variables are included in the regressions as covariates of outcome models (Panel A to Panel D) and treatment models (Panel B and Panel C). Robust standard errors in parentheses are clustered at the school level.

* *p* < 0.1,

** *p* < 0.05,

*** *p* < 0.01.

**Table 3:** Balance tests for covariates of propensity score matching.

Variable	Unmatched	Mean		% bias	% reduct	*t*-test	
	Matched	Treated	Control		|bias|	*t*-statistics	*p*>|t|
Gender	U	0.536	0.522	2.7	14	0.71	0.476
	M	0.535	0.547	-2.3		-0.61	0.543
Age	U	14.598	14.635	-4.9	48.5	-1.29	0.197
	M	14.596	14.577	2.5		0.68	0.497
*Hukou*	U	0.62	0.624	-1.0	-269	-0.26	0.799
	M	0.619	0.602	3.6		0.93	0.354
Boarding	U	0.182	0.421	-53.9	94.9	-14.14	0.000
	M	0.182	0.194	-2.8		-0.81	0.416
Only child	U	0.327	0.361	-7.3	64.6	-1.92	0.055
	M	0.327	0.315	2.6		0.69	0.493
Family economic status (ref: very poor)							
Poor	U	0.087	0.231	-40.1	97.5	-10.51	0.000
	M	0.086	0.083	1.0		0.33	0.738
Moderate	U	0.787	0.661	28.3	94.7	7.42	0.000
	M	0.789	0.796	-1.5		-0.42	0.673
Rich	U	0.104	0.061	15.4	96.2	4.06	0.000
	M	0.102	0.1	0.6		0.14	0.889
Very rich	U	0.004	0.003	2.6	21.2	0.68	0.496
	M	0.004	0.006	-2.0		-0.45	0.652
Parental education	U	9.665	9.5	5.7	83.2	1.49	0.136
	M	9.671	9.644	1.0		0.26	0.797
Sample	Ps R2	LR chi2	p>chi2	MeanBias	MedBias	B	R
Unmatched	0.084	321.69	0	16.2	6.5	70.7	0.68
Matched	0.001	4.4	0.928	2	2.2	8.1	1.13

*Notes*: B represents absolute standard deviation and R denotes the standard deviation ratio.

Tables 4 and 5 demonstrate the results of sensitivity analysis based on Oster (2019)’s bounds for linear regressions and Rosenbaum’s bounds for the PSM approach, respectively. Table 4 reports the bounding estimates of Oster’s method. Following Botha et al. (2021) [46], we assumed a R_max_ = 1.3(R^2^), where R_max_ represents the goodness of fit when controlling for all observed and unobserved factors associated with child health and R^2^ is from the linear regressions with all controls. The results showed that the lower bounds (absolute value) of the health disparities between migrant and left-behind children were above zero and only if the selection on unobservables is 3.581 times higher than that on the observables would it be possible to eliminate the observed effects. Such bounding results suggested that we could not reject that the association of children’s migration and health indicate at least a partial causal relationship. The Rosenbaum bounds in Table 5 indicated that the health effects of children’s migration remained significant until a hidden bias (gamma) or unobserved heterogeneity increased to 1.1 to 1.4 times. Overall, the sensitivity analysis reveals that our findings are robust against unobserved heterogeneity to a certain extent.

**Table 4:** Robustness to unobserved determinants of child health.

	(1)	(2)	(3)	(4)
	Self-reported health	Sick frequency	HAZ	BAZ
Migrant children	0.168***	-0.035**	0.155***	0.186***
	(0.039)	(0.017)	(0.050)	(0.059)
Bounds: (*β*_0_, *β*_1_)	[0.139, 0.168]	[-0.035, -0.029]	[0.128, 0.155]	[0.163, 0.186]
δ required for *β* = 0	3.581	3.884	4.482	4.708
Controls	Yes	Yes	Yes	Yes
Observations	2759	2757	2728	2678
R^2^	0.043	0.019	0.158	0.028
R_max_	0.056	0.024	0.206	0.036

*Notes*: Robust standard errors in parentheses are clustered at the school level. The lower bound and upper bound of bounds analyses are shown in brackets. The bounds analysis assumes R_max_ = 1.3(R^2^), where R^2^ is from the OLS regressions with all controls. The lower bound *β*_1_ is estimated on the basis that the proportional amount of selection on unobservables to selection on observables is 0 (i.e., δ = 0). The lower bound *β*_0_ is calculated when δ = 1, that is, the proportional degree of selection on unobservables is equal to selection on observables (δ = 1). The estimated δ required for *β* = 0 indicates that relative to selection on observables, there should be δ times the amount of selection on unobservables to become insignificant.

* *p* < 0.1,

** *p* < 0.05,

*** *p* < 0.01

**Table 5:** Rosenbaum sensitivity analysis for the health differences between migrant children and left-behind children.

Gamma	Self-reported health		Sick frequency		HAZ		BAZ	
	sig+	sig-	sig+	sig-	sig+	sig-	sig+	sig-
1	0.000	0.000	0.013	0.013	0.000	0.000	0.000	0.000
1.1	0.000	0.000	0.079	0.001	0.000	0.000	0.000	0.000
1.2	0.000	0.000	0.125	0.000	0.000	0.000	0.000	0.000
1.3	0.002	0.000	0.254	0.000	0.013	0.000	0.000	0.000
1.4	0.045	0.000	0.29	0.000	0.143	0.000	0.015	0.000
1.5	0.269	0.000	0.512	0.000	0.5	0.000	0.133	0.000

*Notes*: gamma: log odds of differential assignment due to unobserved factors; sig+: upper bound significance level; sig-: lower bound significance level

### Heterogeneity by hukou type

**Fig 2:**
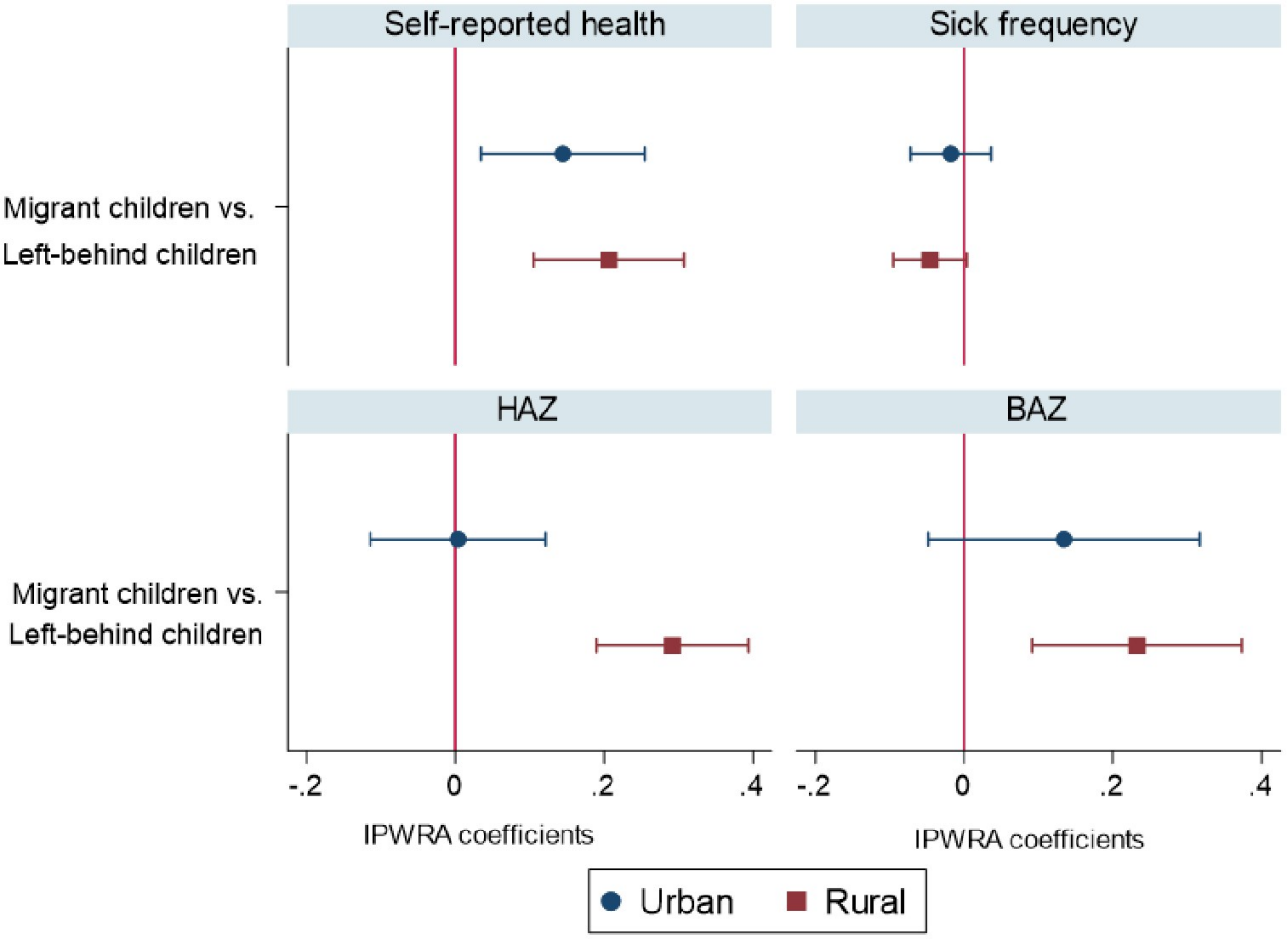
IPWRA estimates for urban and rural subsamples.

Given the differences in socioeconomic development between urban and rural areas, we further conducted a disaggregated analysis by *hukou* type. As shown in Fig 2, for all the four health outcomes, the health disparities between migrant children and left-behind children with a rural *hukou* are larger than their urban peers. Specifically, in comparison with their urban counterparts, migrating with parents has stronger associations with rural children’s self-reported health (β = 0.235, *p<* 0.01 vs. β = 0.172, *p<* 0.05), sick frequency (β = -0.134, *p<* 0.05 vs. β = -0.047, *p* = 0.516), HAZ (β = 0.272, *p<* 0.01 vs. β = 0.009, *p* = 0.957), and BAZ (β = 0.247, *p<* 0.01 vs. β = 0.145, *p* = 0.150). One major possible reason is that rural left-behind children are often more disadvantaged than urban ones in terms of family economic status, parental care, and social support [27, 30]. As such, in comparison with being left behind, rural children benefit more in health when migrating with parents to cities.

### Mechanism analysis

To provide insight into our main findings, we explored possible channels behind the health effects of children’s migration, including parental care, family relationships, peer relationships, and teacher discrimination (see details of the indicators in Table 6). In terms of parental care, the IPWRA estimates suggested that children’s migration positively predicted parental life care (β = 0.199, *p<* 0.01), study care (β = 0.174, *p<* 0.01) and mind care (β = 0.116, *p<* 0.01). For family relationships, the results demonstrated significant and positive associations of internal migration with mother-child relationship (β = 0.099, *p<* 0.01), father-child relationship (β = 0.111, *p<* 0.01) and relationship between parents (β = 0.140, *p<* 0.01). The results in Table 7 also showed that migrant children suffered from more significant teacher discrimination (β = 0.040, *p<* 0.05) compared with their left-behind counterparts. Nevertheless, they had significantly better classmate relationship (β = 0.094, *p<* 0.01) and schoolmate relationship (β = 0.099, *p<* 0.01). To sum up, although migrant children were more likely to face the challenge of teacher discrimination in comparison with their left-behind peers, the higher levels of parental care, family relationships, and peer relationships indicate candidate pathways underlying the positive association between internal migration and health among migrant and left-behind children.

**Table 6:** Details of dependent variables in mechanism analysis.

	Dependent variable	Question descriptions	Variable scores
Parental care	Life care	How often do you go out to watch movies, shows, sports games, etc. with your parents?	Never = 1; Once a year = 2; Once every half year = 3; Once a month = 4; Once a week = 5; More than once a week = 6.
	Study care	Did your parents help you with your homework last week?	No one helped = 1; One or two days = 2; Three or four days = 3; Almost every day = 4.
	Mind care	How often do your parents discuss things happened at school?	Never = 1; Sometimes = 2; Often = 3.
Family relationships	Mother-child relationship	How is the general relationship between you and your mother?	Not close = 1; Not too close nor too far = 2; Very close = 3.
	Father-child relationship	How is the general relationship between you and your father?	Not close = 1; Not too close nor too far = 2; Very close = 3.
	Relationship between parents	Do your parents get along very well?	Yes, they do = 1; No, they don’t = 0.
Peer relationships and teacher discrimination	Nice classmates	How much do you agree with the following statements about your school life: Most of my classmates are nice to me?	Strongly disagree = 1; Somewhat disagree = 2; Somewhat agree = 3; Strongly agree = 4.
	Close schoolmates	How much do you agree with the following statements about your school life: I feel close to people in this school?	Strongly disagree = 1; Somewhat disagree = 2; Somewhat agree = 3; Strongly agree = 4.
	Teacher discrimination	Do you think the school teachers are prejudiced against students from non-local county/district?	Not prejudiced at all = 1; A little bit prejudiced = 2; Somewhat prejudiced = 3; Very prejudiced = 4.

*Notes*: All items were reported by students except for teacher discrimination, which was reported by students’ parents due to the lack of information about the corresponding student-reported question.

**Table 7:** IPWRA estimates for possible mechanisms.

*Panel A*: *Parental care*	(1)	(2)	(3)
	Life care	Study care	Mind care
Migrant children	0.199***	0.174***	0.116***
	(0.042)	(0.042)	(0.024)
*N*	2761	2750	2763
*Panel B*: *Family relationships*	(4)	(5)	(6)
	Mother-child relationship	Father-child relationship	Relationship between parents
Migrant children	0.099***	0.111***	0.140***
	(0.023)	(0.025)	(0.016)
*N*	2750	2756	2723
*Panel C*: *Peer relationships and teacher discrimination*	(7)	(8)	(9)
	Nice classmates	Close schoolmates	Teacher discrimination
Migrant children	0.094***	0.099***	0.040**
	(0.033)	(0.037)	(0.019)
*N*	2776	2763	2636

*Notes*: All control variables are included in the regressions as covariates of outcome models and treatment models. Robust standard errors in parentheses. The details of the dependent variables were presented in Table 4.

* *p* < 0.1,

** *p* < 0.05,

*** *p* < 0.01

## Discussion and conclusion

Although a larger number of previous studies have examined the association between parental migration and child health, empirical evidence regarding the link between children’s internal migration and health remains scarce. Using data from the China Education Panel Survey (CEPS) in the academic year 2014~2015, this study examined the disparities in self-reported health, sick frequency, HAZ, and BAZ between migrant children and left-behind children in China. In our empirical analysis, we employed the ordered probit model and OLS estimation as our benchmark regression approaches. Taking possible self-selection bias into account, we used inverse probability weighting with regression adjustment (IPWRA) and propensity score matching (PSM) methods as robustness checks for our main findings. To address the concern of unobserved confounding factors, we applied Oster’s bounds analysis for linear regressions and Rosenbaum bounds analysis for PSM estimation. Besides, we used children’s migrant status in the previous survey (lagged independent variable) as the key independent variable to address the concern of possible reverse causality problem. Our study also investigated the urban-rural heterogeneity of the health effects of internal migration by children’s *hukou* type. In addition, we further explored the possible channels underlying the associations of internal migration with health among migrant and left-behind children.

The empirical results suggest that migrating with parents significantly improves children’s health status (higher self-reported health, HAZ, BAZ, and lower frequency of sickness) in comparison with being left behind. These results were robust after accounting for potential self-selection bias, unobserved heterogeneity, and reverse causality issues. These findings were also in line with the first hypothesis of this study. Further, the health consequences of children’s migration were shown to be more salient for rural children than urban ones, which was consistent with our second hypothesis and some past research regarding the urban-rural socioeconomic gap [47, 48]. In terms of mechanism analysis, despite experiencing increased teacher discrimination, migrant children had higher levels of parental care, family relationships, and peer relationships relative to left-behind children. The results revealed that the third hypothesis proposed by this study cannot be rejected, which suggested that improved parental care, family relationships, and peer relationships could be major reasons that internal migration was positively associated with child health.

Previous studies indicated that children of Chinese internal migrants were more disadvantaged in education and health in comparison with native children living with their parents [14]. The findings of our study further suggested that left-behind children were more prone to nutrition and health risks than migrant children. In this regard, our study highlights the adverse health consequences of parental absence in China. Policymakers should take actions to reduce the occurrence of involuntary parent-child separation due to institutional constraints. Evidence-based policy interventions such as equalization of health resources and services should also be implemented to foster the development of disadvantaged children.

This study has several limitations. First, due to the data constraints, the observations of our study were junior high school students rather than children of all ages, which suggested that the findings of this study should be interpreted and generalized with caution. Second, although we used four health indicators to measure children’s health outcomes, they may not be able to cover all health dimensions, especially for psychological health measures such as emotional stability and depression. Third, the potential mediators we proposed and empirically examined may not fully explain the associations of internal migration with health among migrant and left-behind children. More relevant studies are needed to contribute to the comprehensive evaluation of the impacts of migration on children’s well-being in China as well as other developing countries.
